# Advances on Plant Ubiquitylome—From Mechanism to Application

**DOI:** 10.3390/ijms21217909

**Published:** 2020-10-24

**Authors:** Dongli He, Rebecca Njeri Damaris, Ming Li, Imran Khan, Pingfang Yang

**Affiliations:** 1 State Key Laboratory of Biocatalysis and Enzyme Engineering, School of Life Sciences, Hubei University, Wuhan 430062, China; hedongli@hubu.edu.cn (D.H.); njerirebecca09@gmail.com (R.N.D.); limit@wbgcas.cn (M.L.); 2 Department of Basic and Translational Sciences, School of Dental Medicine, University of Pennsylvania, Philadelphia, PA 19014, USA; drimran@upenn.edu

**Keywords:** ubiquitylation, plant, method, machinery, function, crosstalk, database

## Abstract

Post-translational modifications (PTMs) of proteins enable modulation of their structure, function, localization and turnover. To date, over 660 PTMs have been reported, among which, reversible PTMs are regarded as the key players in cellular signaling. Signaling mediated by PTMs is faster than re-initiation of gene expression, which may result in a faster response that is particularly crucial for plants due to their sessile nature. Ubiquitylation has been widely reported to be involved in many aspects of plant growth and development and it is largely determined by its target protein. It is therefore of high interest to explore new ubiquitylated proteins/sites to obtain new insights into its mechanism and functions. In the last decades, extensive protein profiling of ubiquitylation has been achieved in different plants due to the advancement in ubiquitylated proteins (or peptides) affinity and mass spectrometry techniques. This obtained information on a large number of ubiquitylated proteins/sites helps crack the mechanism of ubiquitylation in plants. In this review, we have summarized the latest advances in protein ubiquitylation to gain comprehensive and updated knowledge in this field. Besides, the current and future challenges and barriers are also reviewed and discussed.

## 1. Introduction

Plants are constantly exposed to dynamic environmental conditions due to their sessile nature, which compels their cells to evolve and acquire the ability to change and survive from their endogenous status rapidly. The internal signal transduction ultimately induces modulation of cellular proteins in response to external stimuli (e.g., light or temperature stress). These post translational modifications (PTMs) impact protein’s location, stability and activity, eventually triggering a faster response [1]. PTMs greatly modify the classic central dogma and thus attract considerable attention. Therefore, PTMs have become a major concern in protein research, especially the reversible PTMs, such as phosphorylation, ubiquitylation, acetylation, methylation and O-GlcNacylation. To date, there are more than 660 PTMs reported in the Uniprot database http://www.uniprot.org/docs/ptmlist) [2]. Particularly, the frequent PTMs crosstalk within or between proteins forwards the signaling cascades accurately in a mandatory manner [3,4]. 

Ubiquitylation is one of the most prevalent PTMs, which was originally identified as a modulator of cellular protein turnover and homeostasis [5]. However, with the advancement of technology, its functions have been extended far beyond what was initially known. Henceforth, ubiquitylation is found widely involved in pivotal processes such as protein turnover, genomic integrity, signaling processing and cell transport to direct cell proliferation, differentiation or survival through communication systems [6,7]. Increasing evidence indicates that ubiquitylation participates in almost all events in the entire life cycle of the plant from seed germination to flowering, senescence, as well as the pathogen responses among others. For instance, during rice seed germination, He et al. (2020) detected 2576 lysine ubiquitylated (Kub) sites in 1171 proteins and the ubiquitylation was supposed to modulate the protein function more than just providing 26S degradation signals in the early stage of rice seed germination [6]. Guo et al. (2017) identified 3263 Kub sites in 1611 proteins in the ethylene treated Petunia Corollas and the global proteome and ubiquitylome were negatively correlated, indicating the involvement of ubiquitylation in protein degradation of petunias petal senescence [8]. Wang et al. (2020) quantified 1926 unique ubiquitylation sites corresponding to 1053 proteins in the de-etiolating seedling leaves of *Zea mays*, reflecting the role of ubiquitylation in photosynthesis and light signaling [9].

Ubiquitylation is second to glycosylation, for having the most complex modifications due to its diverse target-binding ways. This has created great challenges in the identification of the primary modification and functional elucidation. However, the small ubiquitin (Ub) protein with only 76 amino acid is highly conserved throughout all eukaryotic cells, which has formed a universal language in organisms from yeast to humans [7]. This review will discuss the updates on protein ubiquitylation, from its coding mechanism to research methods, functions, crosstalk and related databases. We will also highlight the recent applications of ubiquitylation in the plant kingdom.

## 2. The Ubiquitylation Machinery and Code

**Figure 1:**
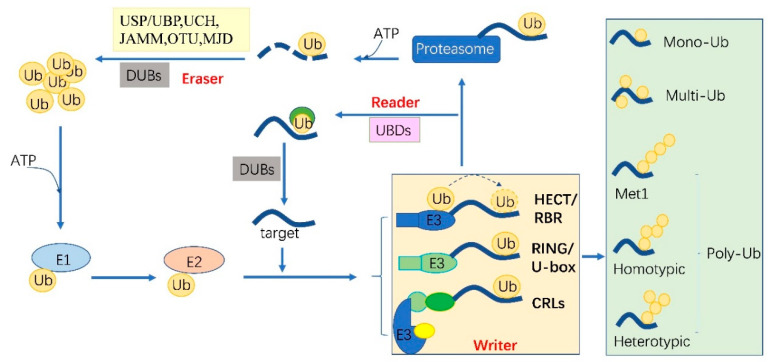
The protein ubiquitylation cascade and its components. Free Ub (Ub) molecules are activated through 3 sequential reactions catalyzed by a Ub-activating enzyme (E1), Ub-conjugating enzyme (E2) and Ub ligase (E3) in an ATP-dependent manner. Based on the transferring mode of Ub Figure 2, the E3s (writer of ubiquitylation) are classed into HECT, RBR, RING, U-box and Cullin-RING E3 ligases. Ub linkage can form into mono-, multimono- (multi-) or poly- ubiquitylation. Ubiquitylation sites are recognized by the proteins carrying Ub binding domains (UBDs, reader of ubiquitylation, including the cap of proteasome) and then direct the targets to be recycled by the deubiquitylases (DUBs, eraser, USP/UBP, UCH, JAMM, OTU and MJD in plants) or 26S proteasome-mediated degradation. HECT, homologous to E6-associated protein C-terminus; RBR, RING-in-between-RING; U-box, a modified RING motif without the full complement of Zn^2+^-binding ligands; USP/UBP, ubiquitin-specific proteases/ubiquitin-specific processing enzymes; UCH, ubiquitin carboxyl-terminal hydrolases; OUT, ovarian tumor proteases; JAMM, JAB1/MPN/MOV34 domain associated metalloisopeptidase; MJD, Machado-Joseph family proteins.

Ubiquitylation describes the process of the conjugation of Ub to substrates, which is sequentially catalyzed by a Ub—activating enzyme (E1), a Ub -conjugating enzyme (E2) and a Ub ligase (E3) [10]. Typically, it forms an iso-peptide bond between the C- terminus of Ub and an ε-amino group of a lysine residue of a substrate but it also can be targeted to other amino acids like Cys, Ser and Thr residues [11,12] or a protein’s N-terminus methionine [13]. Glutamic acid (E), aspartic acid (D) and Alanine (A; neutral) were highly enriched around the Kub sites, however, the basophilic residues histidine (H), arginine (R) and lysine (K) were found to be excluded from the adjacent positions [6,8,9]. The flexibility of conjugation dictates the diversity of ubiquitylation. Ub can be attached to a protein at one residue (mono-ub) or multiple residues (multi-ub), more Ub molecules might be added by E2s/E3s and form polymeric chains (poly-Ub, Figure 1) through selective conjugation to its seven lysine residues (K6, K11, K27, K29, K33, K48 and K63) as well as its N-terminal methionine (M1) [14]. This results in distinct structures and functions. 

The essential Ub are usually encoded redundantly in the eukaryotic genome with mono-Ub unit fusing to ribosomal or organized as poly-Ub units in a tandem linear [15]. These Ub proteins contain 1–7 Ub units. In Arabidopsis, 12 functional Ub have been identified [16]. These fused- or poly-Ub are initially processed into a single Ub molecule by deubiquitylases (DUBs) before being conjugated to its substrates. The DUBs are also responsible for recycling Ub from the substrates (Figure 1). In plants, there are five different DUB families [17]. Approximately 50 DUBs have been identified in Arabidopsis [17] and 100 of these in human beings [18].

Ubiquitylation and deubiquitylation are tightly regulated in vivo. First, the ATP dependent E1 enzyme captures the Ub through its active-site Cys residue and forms a thioester bond between the C terminus of Ub; then the Ub is transferred onto a Cys residue of E2 [19]. E1 and E2 are relatively conserved in eukaryotes. However, the E3 ligases are diverse among organisms and in different biological processes, where they can selectively recruit specific targets. In humans, there are about 9 E1s and 40 E2s [10] and 600 E3s [18]. These enzymes are more complex in plants. Arabidopsis has 2 E1s and 47 E2s, while approximately 1500 potential E3s; Rice has 6 E1s, 49 E2s and more than 1300 E3s [20,21]. E3 ligases are characterized as ubiquitylation writers with different domains, such as HECT(homologous to E6-associated protein C-terminus), RING, UBOX(a modified RING motif without the full complement of Zn^2+^-binding ligands), RBR(RING-in-between-RING) and so forth [22]. The ubiquitylated protein can be read by proteins with Ub- binding domains (UBDs, such as Ub-interacting motif (UIMs) and proteasomal receptor) and then be directed to the downstream biological process. Finally, the DUBs or the regulatory cap of the proteasome will erase the Ub from the substrates, thereafter, the free Ub can be recycled [23,24]. The whole cycle constitutes a powerful ubiquitylation language and performs essential signaling functions in all eukaryotes (Figure 1).

## 3. Methods of Ubiquitylation Detection and Application in Plant Ubiquitylome

The low site-specific stoichiometry, short lifespan, reversible modification, condition-specific expression and complex Ub conjugation architectures bring considerable obstacles in developing deep and accurate catalogs of ubiquitylation. Despite these challenges, greater improvement has been achieved in the identification and verification methods in recent years, especially in plants. 

**Figure 2:**
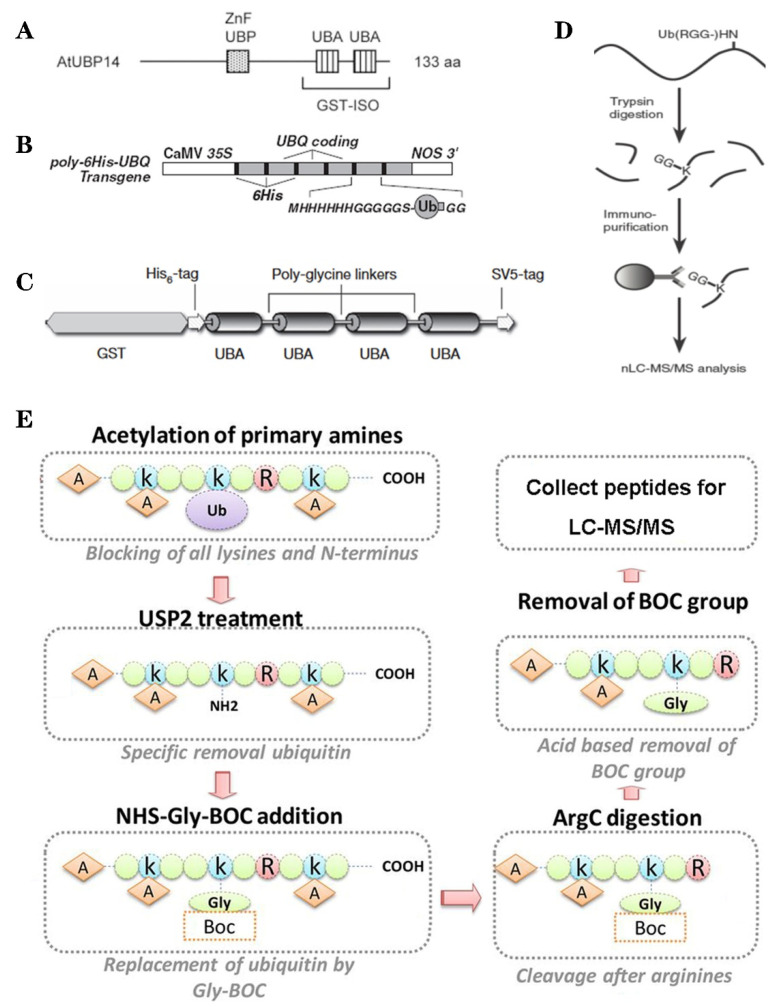
The classic methods of ubiquitylome in plant. (**A**), a single enrichment step approach using UBA (Ub-associated) motif [25]. (**B**), tandem affinity purification (TAP) approach using poly His-tag-UBQ (Ub) motif [26]. (**C**), two-step affinity tandem Ub-binding entities (TUBE) [33]. (**D**), Affinity chromatography using Lys-ε-Gly-Gly (K-ε-GG) specific antibody [29]. (**E**), the Ub COFRADIC (combined fractional diagonal chromatography) pipeline [34].

Based on the binding properties between UBDs protein and ubiquitin, three classic methods have been developed to purify the ubiquitylated proteins, including the single-step enrichment, Tandem Affinity Purification (TAP) protocol and two-step affinity tandem Ub binding entities (TUBEs, Figure 2A–C). The single-step enrichment was established to purify the ubiquitylated proteins using affinity matrices through UBDs and the monoclonal anti-ubiquitin antibodies, directly [25]. This method has successfully identified hundreds of ubiquitylated proteins but many false positives were also identified due to non-specific binding under the nondenaturing conditions. TAP protocol greatly avoid this shortcoming, with an initial production of a stable Arabidopsis transgenic line expressing poly-UBQ gene encoding Ub monomers N-terminally tagged with hexahistidine and then purified with sequential Ub-affinity and strong denaturing nickel chelate-affinity chromatography [26]. Saracco et al. (2009) reported that although only 54 non-redundant targets expressed by 90 possible isoforms were identified by mass spectrometry due to the high stringency of TAP, the accuracy was highly improved. Two-step affinity took advantage of the same Arabidopsis transgenic line of TAP, adopting TUBEs developed by Lopitz-Otsoa et al. [27], which drastically improved the purification stringency and yielded about 950 ubiquitylation substrates in the whole Arabidopsis seedlings [28].

The above methods brought great leap for ubiquitylation identification in plants but have not revealed the exact modified site, through site-directed mutagenesis of the lysine residues, therefore, few ubiquitylated sites have been verified [28]. For the lysine ubiquitylation, when the modified protein is digested by trypsin, the remains becomes a specific C-terminal remnant of Lys-ε-Gly-Gly (K-ε-GG, DiGly). Searching spectra for the typical DiGly footprint, Maor et al. (2007) successfully identified 85 precise DiGly footprints on 56 proteins in Arabidopsis [25]. The development of antibodies that recognize DiGly remnant was the first breakthrough that made the proteome-wide investigation of the exact ubiquitylation sites by LC-MS/MS possible (Figure 2D) [29]. Through affinity chromatography with K-ε-GG specific antibody, the tryptic ubiquitylated peptides were efficiently enriched, thereafter, analyzed by high quality MS/MS, which paved a way for real ubquitylome and as a result, thousands of proteins were identified in different species [6,30,31]. K-ε-GG antibody can also unbiasedly recognize the epitope on the Ub itself and provide the information for poly-linkage sites. However, the K-ε-GG antibody cannot capture modifications occurring at the N-terminal or other residues, moreover, neither can it differentiate the tryptic cleavage of other small related protein modifiers [32], such as small Ub-related modifier (SUMO). 

Ub combined fractional diagonal chromatography (COFRADIC) method was established as a complementary alternative to the K-ε-GG antibody [35]. This protocol first blocks all primary amines (lysines and N termini) via chemical acetylation and removes Ubs with a plant specific DUB USP2cc, then attaches a chemical handle to these free primary amines, subsequently isolating peptides via two consecutive reverse-phase HPLC (RP-HPLC) runs. Afterwards, the handle is removed by ArgC that cleaves the sequence after arginine. As USP2cc specifically recognizes the last five amino acids [36], COFRADIC can successfully avoid the false positive with other Ub-like modifications. USP2cc only recognizes Ub independent of other affected residues, allowing the identification of ubiquitylation on other residues in addition to lysine. Walton et al. (2016) used COFRADIC to identify 16 proteins with N-terminal ubiquitylation in Arabidopsis (Figure 2E) [34]. However, due to the biochemistry-associated bias, COFRADIC generates peptides that are probably too long or too short to be identified by MS/MS and that are difficult for branched Ub chains detection. Therefore, combining the K-ε-GG antibody affinity and the COFRADIC method may provide a deeper insight into ubquitylome.

When a Ub is added to the N-terminal methionine (M) of another Ub, it forms a linear poly-Ub chains that is not detectable by all the above methods. This is because the KGG- antibody does not recognize the characteristic GGMQIFVK peptides while other N-terminally tagged Ub constructs prevent linear poly-Ub chain assembly (e.g., TAP). Kliza et al. (2017) established a new method of identifying linear poly-Ub-modified proteins, in which a lysine-less internally streptavidin tagged Ub (INT-Ub.7KR) was first constructed, followed by stable isotope labeling of amino acids in cell culture (SILAC)-based mass spectrometry [37]. Using this method, several known linear poly-Ub targets were successfully validated in T-REx HEK293T cells, which provided an effective strategy for liner poly-Ub detection. This method could be modified and be applied in plant proteomics research in the future. 

Thanks to the remarkable development of the LC-MS/MS for proteome analysis with high sensitivity and resolution, it is now possible to quickly identify and quantify ubiquitylated proteins in high throughput. Precise relative quantification of ubiquitylated peptides and sites is a big challenge. SILAC has been successfully used for comparison of ubiquitylation dynamics in animal cells. However, its application is rather difficult in plants due to the low efficiency of the in vivo protein labelling. The optimized label-free methods have been proven to be effective in some plants such as petunias, rice and maize [6,8,9], although the accuracy and repeatability require further improvements. In plants, comparative proteome, the advanced tag label methods, for example, isobaric tags for relative and absolute quantitation (iTRAQ) or tandem mass tag (TMT), have been widely used but the K-ε-GG antibody cannot recognize the di-Gly remnant when its N-terminus is derivatized with iTRAQ or TMT. Rose et al. (2016) labelled the ubiquitylated proteins with TMT10 after elution with K-ε-GG antibody and 9000 ubiquitylated peptides were quantified using up to 7 mg labelled sample [38]. To improve the sensitivity and throughput, Namrata et al. (2016) recently developed a rapid and multiplexed protocol termed UbiFast, in which the K-ε-GG antibody is first labelled with TMT and then used to isolate the ubiquitylated peptides. UbiFast facilitated quantification of 10,000 ubiquitylation sites from only 500 μg peptides, which makes large scale comparative ubquitylome more accurate and sensitive [39].

## 4. Multiple Functions Played by Protein Ubiquitylation in Plants 

**Figure 3:**
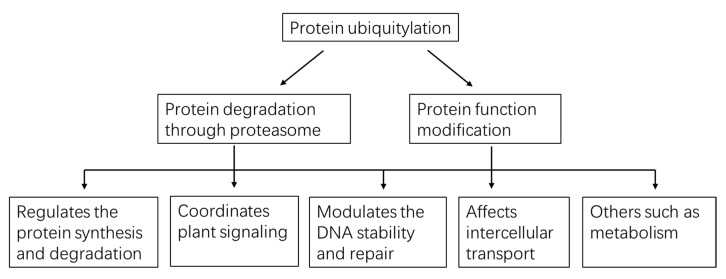
A global view of functions played by protein ubiquitylation in plants.

Using the protein/peptide affinity plus high-quality MS/MS technology, thousands of ubiquitylated substrates can be identified with one experiment. These substrates carry different types of Ub-chain and may possibly exert diverse effects on the targets (Table 1, Figure 3). Mono-Ub/multi-Ub may precisely change the protein activity and interaction [40], notably, mono-ub-dependent protein degradation has recently been reported. Poly-Ub performs diverse functions, with homotypic K48-linked poly-Ub being the predominant (>50%) linkage and mainly guides proteins to the ubiquitin proteasome system (UPS) for degradation [41]. Also, some K11-, K27-, K29- and K33-linked poly-Ub direct proteins for degradation [42]. In Arabidopsis cell-free system, the K29-chain were confirmed to regulate the degradation of DELLA proteins, repressors in GA signaling pathways [43]. K63-linkage is another abundant poly-Ub despite it being proteasome-independent. K63-linked poly-Ub chain formation is critical for vacuolar targeting of the auxin efflux carrier component 2 (PIN2) [44]. Along with the development of the specific antibody, some heterogenous poly-Ub chains were also discovered, such as K63- poly-Ub that may serve as a “seed” for K48-poly-Ub and form the K48/K63 branched chains, which then directed the targets for degradation [45]. However, to date, the evidence of branched poly-Ub chains in plants is not discovered. In a study conducted by Swatek et al. (2019), they demonstrated that the leader protease (Lbpro) of foot-and-mouth disease virus can incompletely remove ubiquitin from substrates and generate DiGly-modified proteins. This Ub-clipping methodology may provide new insight into the combinatorial complexity and architecture of the ubiquitin (including the mono-, multi- or poly- Ub) code in plants [46]. 

### 4.1. Ubiquitylation Regulates Protein Synthesis and Degradation

Ribosomes are the main molecular machinery that generates nascent polypeptide within the cells. The cytoplasmic ribosomes consist of two sub units (40S and 60S) with folded ribosomal RNA (5.8S, 18S and 28S rRNA), approximately 80 ribosomal proteins (RPs) are present in eukaryotes. During the ribosomal synthesis, RPs are initially incorporated into the ribosome in accordance with the processing of pre-rRNA transcripts into mature rRNAs before being exported to the cytoplasm. In this highly efficient process, many RPs are “unemployed” to become potential ubiquitylation substrates for nuclear proteasome-mediated degradation [47]. Stress conditions may cause massive amounts of RPs to become “unemployed” and ubiquitylated and this has been confirmed in de-etiolating maize seedlings and senescing petunias petal [8,9].

Ubiquitylation distinguishes itself from other PTMs as it is widely involved in protein stability. The bulk of protein degradation in living cells depends on UPS. The proteasome complex constitutes of a 14-subunit 20S core protease and an 18-or-more subunit 19S regulatory particle, which can recognize ubiquitylated proteins and degrade them into small peptides, while removing the Ub for recycling [48]. Protein ubiquitylation performs important function of protein’s quality control in cells, clearing those misfolded or damaged proteins, which occurs through tagging substrates with K11-, K48- or K11/K48-branched poly-Ub chains [49]. After treating Arabidopsis seedlings with proteasome inhibitor MG132, more than half of the ubiquitylated proteins increased at the ubiquitylation level, which might be later degraded by 26S proteasome [28]. At the same time, 26S proteasome can be modified by ubiquitylation and the abundance of most ubiquitylated 26S subunits increased significantly (average of 3.9-fold). This increase can be attributed to turnover of inhibited proteasome complexes [28]. Besides, other UPS components, including E1, E2 and E3 enzymes and de-ubiquitylating enzymes were also found ubiquitylated, possibly repressing UPS activity [6,50]. 

Autophagy is another foremost mechanism of protein degradation and remobilization, which maintains cellular homeostasis by recycling cytoplasmic components through forming a double-membrane autophagosome that subsequently degrades them via the lysosome. It is a highly conserved cellular process prevailing in all eukaryotes [51]. Series of autophagy-related proteins (ATGs) have been discovered in plants, so far. In Arabidopsis, the ATG1–ATG13 kinase complex are key positive regulators to induce the autophagic vesiculation [52]. The ubiquitylation system has been reported to control the protein stability of ATG1–ATG13 complex [53]. Qi et al. (year) discovered the RING-type E3 ligases seven in absentia of Arabidopsis thaliana (SINAT) proteins regulate autophagy by interacting with ATG proteins and modulating the stability of the ATG1–ATG13 kinase complex under either normal nutrient or some starvation conditions [54]. During the senescence of petunias petal, ubiquitylation of ATG8b (Lys-11) was first found to be up-regulated by ethylene [8]. Ubiquitylation is also known for the removal of misfolded or aggregated proteins in autophagy. In another study, the NBR1 (neighbor of BRCA1) protein was reported as an adaptor protein recruited to Ub-positive protein aggregates and degraded by autophagy in the animal cells [55].

### 4.2. Ubiquitylation Coordinates Plant Signaling

Because of its reversible nature, rapid kinetics and the versatility of outcomes, ubiquitylation can facilitate the coordination of external and internal environmental signals in space and time. In an earliest study, Shanklin et al. (1987) reported that ubiquitylation mediated the conversion of phytochrome forms between red light-absorbing form (Pr) and far-red light-absorbing form (Pfr.) [64]. The light was then revealed to induce degradation of phytochrome interacting factor PIF1 by the CUL4^COP1–SPA^ E3 ligase initiated photomorphogenesis [65]. Thereafter, the involvement of ubiquitylation in various plant signaling pathways has been broadly reported such as calcium signaling, 14-3-3 proteins and G-proteins mediated signaling [66,67]. 

The proteasome-mediated degradation acts as central regulator in most phytohormone signaling pathways in plants, such as gibberellic acid (GA), abscisic acid (ABA), auxin, brassinosteroids (BRs), ethylene, salicylic acid (SA), jasmonic acid (JA) among others. DELLA (SLR1) protein, one known repressor of the GA signaling, can be degraded by the 26S proteasome via SCF^GID2^ in a GA-dependent manner [43]. In Arabidopsis, the negative regulator *ABI3*-interacting protein2 (AIP2) of ABA signaling can promote abscisic acid insensitive 3 (ABI3) polyubiquitylation for degradation [68], while RING-type E3 ligase keep on going (KEG) can ubquitinate ABI5, the upstream transcriptional factor of ABI3 and regulate its abundance [69]. Auxin or indole-3-acetic acid (IAA) can trigger nuclear cascades by modulating the recruitment of most short-lived AUXIN/INDOLE-3-acetic acid (AUX/IAA) transcriptional repressors through multimeric SCF-type E3 Ub ligases, which then leads to ubiquitylation and proteasome-dependent degradation of AUX/IAA [57,70]. The membrane-bound receptor complex BRI1/BAK1 of Brassinosteroids (BRs) is regulated by K63 poly-ubiquitylation [71]. The ethylene insensitive2 (EIN2) plays a central role in the signaling of gaseous hormone ethylene. This process is tightly regulated through proteasomal degradation [72]. In the absence of ethylene, EIN3 is targeted for degradation by Cullin1-RING Finger E3 Ligase CRL1s [73]. 

### 4.3. Ubiquitylation Modulates DNA Stability and Repair

Sessile plants may encounter serious genotoxic stress capable of evoking complex DNA damage response (DDR) to protect genomic integrity. Ub-dependent signaling could regulate the DDR like double-strand break response (DSB), nucleotide excision repair (NER), together with other PTMs. During template switching and the DSB response, K63 Poly-Ub assembles as a scaffold for the signaling complex. The BRCA1/BARD1 Ub ligase was observed to assemble K6 linkages in vitro and in overexpression systems. However, a detailed mechanism between K6 linkages and DDR is still elusive [74]. K6- and K33-linked polyubiquitylation were detected having undergone a bulk increase in response to DNA damaged by UV [75]. Proliferating cell nuclear antigen (PCNA) can recruit DNA trans lesion polymerases or the translocase for DDR [76]. Upon rice seed imbibition, Kub164 of PCNA was induced significantly, meanwhile, four Rad23 (Radiation) family proteins and a DDI1(DNA-damage inducible) were rapidly ubiquitylated, which is evidence for widespread DDR during the initiation of seed germination [6].

Monoubiquitylation plays a central role in modulating nucleosome/chromatin structure (histones H2A, H2B, H3 and H4) and DNA accessibility for gene-specific transcription [77,78,79]. Histone H2A was the first protein to be identified as a substrate for ubiquitylation [80]. In human cells, about 10% of H2A molecules are monoubiquitylated on K119 by a family of multi-subunit E3 ligases known as poly-comb repressive complex 1 (PRC1) [81]. H2A monoubiquitylation can induce repressive histone H3K27 trimethylation and in turn recruits more PRC1 complexes, which allows H2A ubiquitylation to spread along whole chromosomes and further restricts gene expression [82]. Beside inhibiting transcription, monoubiquitylation of other histones result in divergent consequences in controlling gene expression, DNA methylation or DNA repair, for example ubiquitylation of K120 on H2B stimulates gene expression [83]; monoubiquitylation of proximal Lys residues on H3 enables epigenetic inheritance of DNA methylation status [84]; monoubiquitylation of K91 of H4 is important for DNA damage signaling [85]. De-ubiquitylation on these histones occurring at the right time and place promotes rapid changes in transcriptional programs [86].

### 4.4. Ubiquitylation Affects Intercellular Transport

Ubiquitylation has an extended role in controlling cellular transmembrane transport, protein trafficking, precise location and protein stability. Epidermal growth factor receptor (EGFR) is the first protein identified as evidence of ubiquitylation role in protein transport. This modification occurs on multiple Lys residues during endocytosis internalization [87]. The endosomal sorting complex required for transport (ESCRT) machinery can traffic the flow of ubiquitylated proteins from endosomes to lysosomes. The five distinct complexes (ESCRT-0, -I, -II, -III and the Vps4 complex) have a clear division of tasks to guarantee a smooth transport of ubiquitylated cargos along the endosomal trafficking pathway [88]. The internalized receptors were first recognized by the ESCRT-0 complex, which was associated with multiple Ub subunits and then handed over sequentially to other complexes [89]. Endoplasmic-reticulum-associated protein degradation (ERAD) eliminates defective membrane or luminal proteins of the ER. Substrates of ERAD are ubiquitylated by ER-localized E3s and are delivered to the proteasome for degradation. Membrane-localized E3 ligases are also found in the Golgi apparatus, mitochondria, chloroplast or peroxisomes [6,90]. Ubiquitylation is capable of controlling the trafficking machinery. Contrarily, monoubiquitylation of COPII coat protein controls secretory protein transfer from the ER to the Golgi apparatus [91]. Vesicle transport can move proteins among different locations within the cell organelles. Soluble N-ethylmaleimide-sensitive factor attachment protein receptors (SNARE) are required for the fusion of transport vesicles with the correct target membrane. VAMP8 (v-SNARE) ubiquitylation is required for vesicle fusion in cytokinesis [92]. The authors have identified 2 v-SNARE and 7 t-SNARE proteins that were rapidly ubiquitylated during rice seed germination [6].

## 5. Crosstalk between Ubiquitylation and Other PTMs

Plant proteins are subjected to a wide array of PTMs. These modifications can occur on the same amino acid residue (s) of a substrate at various temporal points or different amino acid residues of the same substrate. Different PTMs influence each other in coordinating multiple signal transduction events in an orchestrated manner. PTM crosstalk greatly improves the ability of sessile plants for rapid response to different external and internal cues. Among these PTMs, several are reversible including ubiquitylation, phosphorylation, SUMOylation, S-nitrosylation and glycosylation, which may endow proteins with opposing biochemical activities. 

As two of the most prevalent PTMs in eukaryotic proteomes, crosstalk between phosphorylation and ubiquitylation can take various forms. For example, phosphorylation can change the stability, function site or substrate recognition of the E3 enzyme and ubiquitylation can directly modify phosphorylation receptor kinase activity [93]. The crosstalk will ultimately cause different outcomes, such as substrate degradation (e.g., FLS2 in Arabidopsis, the leucine-rich repeat receptor-like kinase Flagellin-Sensing 2) [94], changing the enzymatic activity (e.g., plant U-box (PUB) type E3 Ub ligase PUB12/13) [95] or subcellular localization (e.g., BRI1) [71]. Different phosphorylation sites of the same substrate can lead to opposite effects, for PUB2 in Medicago, phosphorylation at Ser316 suppresses the E3 ligase activity of PUB2; however, phosphorylation at Ser421 promotes its E3 ligase activity [96]. The most cited crosstalk between phosphorylation and ubiquitylation is phosphodegrons, in which one or more phosphorylation site(s) function in a short linear motif to promote the subsequent ubiquitylation and degradation of a substrate. The irreversible and robust phosphodegrons are critical for cell cycle progression. The Cdc4 protein was the first F-box protein to be described, which is capable of forming a WD40 fold that recognizes phosphorylated peptides [97]. Using the sequential enrichment method, Swaney et al. (2016) globally analyzed the phosphorylation and ubiquitylation cross-talk in protein degradation in yeast *Saccharomyces cerevisiae*, 466 proteins with 2100 phosphorylation sites co-occurring with 2189 ubiquitylation sites were identified. Further analysis showed that distinct phosphorylation sites are often in conjunction with ubiquitylation in a highly conserved manner [67]. 

In addition to the above-mentioned crosstalk, ubiquitylation widely interacts with many other PTMs. In Arabidopsis, SNC1 is acetylated at the N terminal, functioning as N-degron for its ubiquitylation leading to its degradation, which results in a decrease in plant’s immunity [98]. Redox modifications, for example, S-nitrosylation, disulfides and S-glutathionylation can directly regulate enzymes constituting the ubiquitylation system and affect enzymatic activity [99]. 

Ub itself can be modified by various PTMs. For instance, phosphorylation has been observed in most of Ub serine, threonine and tyrosine residues [100] and the most frequently modified residues are Ser57 and Ser65 in yeast and mammalian cells, respectively. For example, the PINK1 kinase can phosphorylate S65 of Ub at the mitochondrial surface and activate ubiquitin ligase Parkin in mammals, which will promote the ubiquitylation of numerous mitochondrial outer membrane proteins, finally resulting in the mitochondrial autophagy (mitophagy) [101]. Phosphorylation also inhibits certain E2s, E3s and DUBs and modulates the ubiquitylation cycle [102]. Only 0.03 and 0.01% acetylation of Ub occurred on K6 and K48 in human cells. Overexpression of acetyl-mimetic Ub (K6Q) can repress K11-, K48- and K63-linked Ub chain elongation on substrates but stabilize the monoubiquitylation of histone H2B. We also have detected phosphorylated and acetylated Ub in the germinating rice seed [103,104]. These modifications may change the structure of the Ub-chain and its related signaling in vivo.

## 6. Related Databases Developed for Plant Ubiquitylation

Owing to the advancement of high throughput of high-quality mass spectrometry (MS)-based proteomics, numerous ubiquitylation sites have been identified, resulting in an enormous collection of ubiquitylation data. Accordingly, some professional databases have been developed to collect the massive ubiquitylated data and analyze the protein ubiquitylation networks (Table 2).

Keeping in view of the importance, in 2007, Chernorudskiy et al. created UbiProt (http://ubiprot.org.ru) which provided retrievable information about overall characteristics of a particular ubiquitylated protein, related ubiquitylation and deubiquitylation machinery and related literature references. However, this database could only collect 1104 ubiquitylated substrates from 12 species [105]. Considering the expensive and time-consuming nature of experimental methods, a computational method PEIMAN (Post-translational modification Enrichment Integration and Matching Analysis) was developed to study, analyze, predict, count and compute ubiquitylation and other PTMs [106]. Around 2.2% of genomic genes in most species belong to the UPS and thus Du et al. (2009) constructed the plants UPS database (http://bioinformatics.cau.edu.cn/plantsUPS/) that enabled the comparative analysis of UPS in higher plants [20]. This database collected 24 E1,417 E2 and 7624 E3 from seven plant species distributed in 11 UPS-involved gene families. However, due to some reasons, these databases are not updated time to time. Recently, Van-Nui et al. established UbiNet (http://csb.cse.yzu.edu.tw/UbiNet/), which has accumulated 43,948 experimentally verified ubiquitylation sites from 14,692 ubiquitylated proteins of humans and also provides a comprehensive map of intracellular ubiquitylation networks [107]. In Arabidopsis, an ubquitylome using modified COFRADIC technology was shared by the author with information concerning the respective splice variant, the modified sequence, the sequence window, as well as the position of the site within the protein (http://bioinformatics.psb.ugent.be/webtools/Ub_viewer/) [34].

Meanwhile, some databases provide integrative information of multiple PTMs and facilitate analysis of the related crosstalk. Among them, PTMCode (https://ptmcode.embl.de/) provides a resource of known and predicted PTMs functional associations between protein and PTMs within and between interacting proteins, it was updated in 2015 and currently contains 316,546 modified sites from 69 different PTM types [108]. Another database, dbPTM (http://dbptm.mbc.nctu.edu.tw/), an informative resource for PTMs, was established in 2012, now it has been updated to version dbPTM3.0, collecting 908,917 experimental PTM sites of over 130 PTM types. In addition, dbPTM also provides comprehensive functional and structural analyses for PTMs [109]. Patrick et al. (2019) integrated 19 types of protein modifications in plant proteins from five different species and established the Plant PTM Viewer, which comprises approximately 370, 000 PTM sites and remains open for submission, this repository will help to assume the role and potential interplay of PTMs in specific proteins [110]. 

In parallel, several useful softwares were also developed during the establishment of these databases. These include CKSAAP_UbSite (http://protein.cau.edu.cn/cksaap_ubsite/), UbiPred (http://iclab.life.nctu.edu.tw/ubipred) and UbPred (http://www.ubpred.org). These web servers may facilitate the prediction of ubiquitylation sites in proteins according to extract sequence features of amino acids and the class-balanced accuracy reaches over 70%.

## 7. Future Challenges of Plant Protein Ubiquitylation 

Along with the great achievement, more concerns have also been raised in the study of plant protein ubiquitylation and addressing these challenges will bring new insights into the ubiquitylation structure and function. First, the discovery of particular connections of ubiquitylation in plants. Although the linkage-specific antibodies, affinity purification and mass spectrometry provide powerful approaches in investigating the designated Ub modified sites (N-terminal or lysine residues), they cannot be utilized for exploration of specific modification sites (like Cys, Ser and Thr residues). Therefore, as a greater challenge, exponential expansion of knowledge is needed to understand the complex poly-Ub chain structure (homo-/hetero- and branched-/linear- chain) and its corresponding functions. Limited by the relatively long lifespan and complex cell structure, the development and application of the technologies in plants are especially difficult. Moreover, understanding the detailed mechanism of the ubiquitylation system and uncovering its potential functions requires further exploration. Interactions between an E3 ligase and its cognate substrate are weak and dynamic and the ubiquitylation is unstable and reversible, which fail to identify the full spectrum of related E3 ligase and its substrates. Therefore, methods of verification and reconstruction of these modifications in vivo or in vitro are urgently required. The establishment of a comprehensive database is something to achieve in the near future. Recently, databases have been established to collect and process the massive ubiquitylation data, however, many of them are not promptly updated. Extensive integration of existing information (including ubiquitylation and the other PTMs) will provide a reliable public platform for scientific research, which will bring new insights into deciphering the PTM language (not only ubiquitylation) underlying the proteome and facilitate in developing innovative technologies in agriculture.

## Figures and Tables

**Table 1:** Potential function of various ubiquitin linkage (The red ones have not been recorded in plants).

Ub code	Function	Substrate	Reference
Mono-/multi-ubiquitylation	changes the protein activity and interaction	e.g., lysine 119 of H2B	[56]
K29-chains	proteasomal degradation	e.g., DELLA proteins	[43]
K48-chains	proteasomal degradation	e.g., Aux/IAA	[57]
K63-chains	endocytic sorting, DNA repair, degradation but proteasome-independent	e.g., PIN2	[44]
M1	inflammation signaling	e.g., NF- κ B	[58]
K6-chains	Mitophagy	e.g., mitofusin-2 (Mfn2)	[59]
K11-chains	proteasomal degradation/cell cycle regulation	e.g., anaphase-promoting complex APC/C	[60]
K27-chains	proteasomal degradation	e.g., NS4B	[61]
K33-chains	proteasomal degradation	e.g., ERCC1 (nucleotide excision repair protein)	[62]
M1/K63-linked	transcription factor activation (K63-poly-ub as a prerequisite for the formation of M1-poly-Ub)	e.g., canonical I κ B kinase IKK α and IKK β	[63]
K11/K48-linked	proteasomal degradation, cell-cycle and quality control	e.g., anaphase-promoting complex APC/C	[49]
K48/K63-chains	proteasomal degradation (K63- poly-ub serving as a “ seed ” for K48-poly-ub)	e.g., proapoptotic regulator TXNIP	[45]

**Table 2:** Databases Developed for Plant Ubiquitylation.

Name	Website	Aims	Updated Time
UbiProt	http://ubiprot.org.ru	experimentally obtained	2007
PEIMAN	http://bs.ipm.ir/softwares/PEIMAN	predict and compute the ubiquitylation and other PTMs	2015
plantsUPS	http://bioinformatics.cau.edu.cn/plantsUPS	comparative analysis of UPS in higher plants	2009
COFRADIC method	http://bioinformatics.psb.ugent.be/webtools/Ub_viewer	Arabidopsis	2016
PTMCode	https://ptmcode.embl.de	integrative information of known and predicted PTMs	2015
dbPTM	http://dbptm.mbc.nctu.edu.tw	comprehensively functional and structural analyses for PTMs	2019
Plant PTM Viewer	https://www.psb.ugent.be/webtools/ptm-viewer	tools to analyze the potential role of PTMs	2019
CKSAAP_UbSite	http://systbio.cau.edu.cn/cksaap_ubsite/i	software to predict ubiquitylation sites	2013
UbiPred	http://flipper.diff.org/app/tools/info/2503	predict ubiquitylation sites	2010
UbPred	http://www.ubpred.org	random forest-based predictor of potential ubiquitination sites	2010
